# A systematic review on the qualitative experiences of people living with lung cancer in rural areas

**DOI:** 10.1007/s00520-024-08342-4

**Published:** 2024-02-06

**Authors:** Nabilah Ali, David Nelson, Daisy McInnerney, Samantha L. Quaife, Despina Laparidou, Peter Selby, Ros Kane, Sarah Civello, Dawn Skinner, Zara Pogson, Michael D. Peake, Ava Harding-Bell, Samuel Cooke

**Affiliations:** 1 Lincoln Medical School, College of Health and Science, Universities of Nottingham and Lincoln, Lincoln, LN6 7TS UK; 2 College of Health and Science, Lincoln International Institute for Rural Health, University of Lincoln, Lincoln, LN6 7TS UK; 3 Macmillan Cancer Support, London, SE1 7UQ UK; 4 Centre for Cancer Screening, Prevention and Early Diagnosis, Wolfson Institute of Population Health, Queen Mary University of London, London, EC1M 6BQ UK; 5 Community and Health Research Unit, School of Health and Social Care, University of Lincoln, Lincoln, LN6 7TS UK; 6 School of Medicine, University of Leeds, Leeds, LS2 9JT UK; 7 School of Health and Social Care, University of Lincoln, Lincoln, LN6 7TS UK; 8 Lincoln County Hospital, United Lincolnshire Hospitals NHS Trust, Lincoln, LN2 5QY UK; 9 Pilgrim Hospital, United Lincolnshire Hospitals NHS Trust, Boston, PE21 9QS UK; 10 Cancer Research UK, London, E20 1JQ UK; 11 Glenfield Hospital, University of Leicester, Leicester, LE1 7RH UK; 12 Swineshead Patient Participation Group, Swineshead Medical Group, Boston, PE20 3JE UK

**Keywords:** Lung cancer, Experiences, Qualitative research, Rural health, Systematic review

## Abstract

**Purpose:**

To synthesize the qualitative literature exploring the experiences of people living with lung cancer in rural areas.

**Methods:**

Searches were performed in MEDLINE, CINAHL, and PsycINFO. Articles were screened independently by two reviewers against pre-determined eligibility criteria. Data were synthesized using Thomas and Harden’s framework for the thematic synthesis of qualitative research. The CASP qualitative checklist was used for quality assessment and the review was reported in accordance with the ENTREQ and PRISMA checklists.

**Results:**

Nine articles were included, from which five themes were identified: (1) diagnosis and treatment pathways, (2) travel and financial burden, (3) communication and information, (4) experiences of interacting with healthcare professionals, (5) symptoms and health-seeking behaviors. Lung cancer diagnosis was unexpected for some with several reporting treatment delays and long wait times regarding diagnosis and treatment. Accessing treatment was perceived as challenging and time-consuming due to distance and financial stress. Inadequate communication of information from healthcare professionals was a common concern expressed by rural people living with lung cancer who also conveyed dissatisfaction with their healthcare professionals. Some were reluctant to seek help due to geographical distance and sociocultural factors whilst others found it challenging to identify symptoms due to comorbidities.

**Conclusions:**

This review provides a deeper understanding of the challenges faced by people with lung cancer in rural settings, through which future researchers can begin to develop tailored support to address the existing disparities that affect this population.

**Supplementary Information:**

The online version contains supplementary material available at 10.1007/s00520-024-08342-4.

## Background

Lung cancer is the second most diagnosed cancer globally, accounting for approximately 2.2 million cases and is the leading cause of cancer mortality [1–3]. In 2020, lung cancer represented approximately one in 10 (11.4%) of all cancer diagnoses and one in five (18.0%) of all cancer deaths worldwide [1]. Smoking remains the primary risk factor for developing lung cancer [4, 5], although other contributors include environmental pollution, occupational exposures, radon exposure, age, gender, race, and pre-existing lung disease [4–6]. Not all people with these risk factors will develop lung cancer and others without any known risk factors will, suggesting that genetic factors play an important role in the etiology of lung cancer [7, 8]. Lung cancer has the widest deprivation gap of all cancers, with people who experience worse socioeconomic deprivation having a higher risk of mortality compared to those from more affluent backgrounds [9]. However, attention is increasingly turning to factors beyond socio-economic deprivation that interact to perpetuate inequities in both lung cancer incidence and survival rates [10].

One factor to consider is the intersectionality between lung cancer and rurality. Whilst there remains no universal definition of “rural,” in the UK, the Department for Environment, Food & Rural Affairs defines areas as “rural” if they have less than 10,000 residents [11]. There is increasing evidence to suggest that people living with lung cancer in rural areas may experience unique inequalities in care and treatment compared to those living in urban areas [12, 13]. Examples include greater treatment delays [14], poorer access to care including preventative services [15], higher incidence rates [16], later stage presentation and diagnosis [16], worse survival rates, and higher overall mortality [17]. Whilst there is clear and substantial epidemiological evidence indicating that people with lung cancer in rural areas experience inequalities, there is a need for a systematic review of published qualitative evidence to better understand patterns of health behaviors, lived experiences, and healthcare needs [18] of rural lung cancer patients. The qualitative evidence generated from this review may enhance quantitative evidence in informing the development of recommendations for potential interventions that may begin to address the unique challenges faced by this population.

This systematic review focuses exclusively on rural areas in high-income countries which we define as those belonging to the Organization for Economic Co-operation and Development (OECD), due to the significant healthcare disparities between high- and low-income countries [19, 20]. This was to enable a comprehensive exploration of experiences of living with lung cancer in rural areas where healthcare infrastructure and resources are comparatively advanced compared to low-income countries. Furthermore, addressing inequalities associated with rurality remains largely absent from cancer health policy in economically developed countries [21] many of which have sizeable rural populations. The aim of this systematic review is to synthesize the qualitative literature exploring the experiences of people living with lung cancer in rural areas. To date, evidence has largely focused on improving the quality of clinical lung cancer services and much less on individual patient experience. This review therefore aims to answer the following question: What are the qualitative experiences of people living with lung cancer in rural areas in OECD countries? This review has the following objectives:

1. To identify and collate evidence surrounding the qualitative experiences of people with lung cancer living in rural areas.
2. To thematically synthesize evidence surrounding the qualitative experiences of people with lung cancer living in rural areas.

## Methods

### Study design

This systematic review was conducted in accordance with the Enhancing Transparency in Reporting the Synthesis of Qualitative Research checklist (ENTREQ) [22] (Supplementary information 1) and the Preferred Reporting Items for Systemic Reviews and Meta-Analyses (PRISMA) (Supplementary information 2). The protocol was registered on the Open Science Framework (https://osf.io/mjyhn/, last updated 08-Dec-2022). The initial idea for the review and design was led on by DN and SC with support from all of the wider team who sat on a project Steering Group.

### Search strategy

The search strategy (Supplementary information 3) was developed by two members of the review team SC and DN. Keyword searches together with Truncation (*) and Boolean operators (OR and AND) were performed in MEDLINE, CINAHL, and PsycINFO by SC on 12-April-2023. Searches of databases were pre-determined as to identify all available evidence. Retrieved records were downloaded and stored in Rayyan software [23] to support management and screening. Titles, abstracts, and full texts were independently screened by NA and SC with DN cross-checking for quality or in the event of any discrepancies. All database searches were supplemented with searches on Google Scholar and the reference lists of included articles. Publication date was limited to between the years 2000 and 2023.

### Eligibility criteria

#### Inclusion

Peer-reviewed qualitative (including mixed methods) studies (in the English language) reporting primary data on the experiences of adults (18 +) living with lung cancer residing in rural, regional, or remote areas of OECD countries were included. Studies reporting on the experiences of people with lung cancer alongside other types of cancer were included but all studies had to explicitly report their setting or sample as “rural,” “remote,” or “regional” to be included. Where studies had both rural and urban samples, only data from the rural, regional, or remote respondents were included.

#### Exclusion

Studies that explicitly focused on lung cancer populations within urban and metropolitan settings or whose study populations were under age 18 years were excluded from this review. Furthermore, studies that provided cancer experience data where it was not definitively clear as to the residence of participants or the cancer type and those conducted in middle- and low-income countries were excluded as were secondary research studies (studies including systematic reviews, editorials, case reports, and opinion pieces).

### Data extraction

Following the identification of relevant articles after title, abstract, and full text screening, data were extracted using an adapted Cochrane Data Extraction Template [24]. The data extracted from each study included as follows: (1) author and year of publication, (2) study setting, (3) aim of study, (4) participants, (5) methods and design, (6) rural setting, (7) summary of key findings. NA extracted all data, with SC and DN cross-checking for accuracy.

### Quality assessment

The quality of included studies was independently assessed by DL and DN using the Critical Appraisal Skills Program (CASP) Qualitative Studies Checklist [25]. Where there were discrepancies over the quality of articles, DL, DN, and SC met to reach agreement on the final decision. This checklist consists of 10 questions that cover rigor, methodology, credibility, and relevance. Some papers used a mixed methods design, in which case the CASP checklist was only applied to the qualitative components.

### Data analysis

Thematic synthesis of the qualitative data was undertaken using Thomas and Harden’s approach to the thematic synthesis of qualitative research in systematic reviews [26]. A thematic synthesis approach was chosen as it provides a flexible, systematic, and transparent method in identifying rich and detailed qualitative data across multiple studies for synthesis [26, 27]. This process involves as follows: (1) inductive line-by-line coding of relevant text; (2) developing “descriptive themes”; and (3) generating “analytical themes.” Initial line by line codes was created in Microsoft Word, then uploaded to the NVivo software system to facilitate the generation of both the descriptive and analytical themes. NA led on the thematic synthesis with iterative input from SC and DN. The development of descriptive themes remains close to the primary research studies that were included in the review, whereas the analytical themes are where the reviewers go beyond the primary studies and generate new interpretive insights or explanations [26]. Clinical members of the team supported the analysis and interpretation of qualitative data.

### Author reflexivity

It is important for researchers conducting qualitative research to understand the assumptions and preconceptions they have which may influence the research process allowing the reader to contextualize the relationship between the researchers and the research [28, 29]. The current research team represents diverse professional backgrounds with a range of clinical and academic expertise. The team includes as follows: NA, a medical student with interest in cancer and rurality; DN and SC, rural health researchers with expertise in cancer survivorship and systematic reviews; DL, a health services researcher with experience in systematic reviews and qualitative analysis; SQ and DM, behavioral researchers with experience in lung cancer screening and cancer lived experience research; ZP, a respiratory consultant and SCi and DS, clinical nurse specialists, all with clinical experience in respiratory and lung cancer care; PS, a professor of cancer medicine with clinical research in oncology and cancer care; RK, a professor of nursing and public health with experience in cancer survivorship and rurality; AH-B, a public contributor with lived experience as a lung cancer caregiver; and MP, an emeritus consultant and honorary professor of respiratory medicine.

## Results

**Fig. 1:**
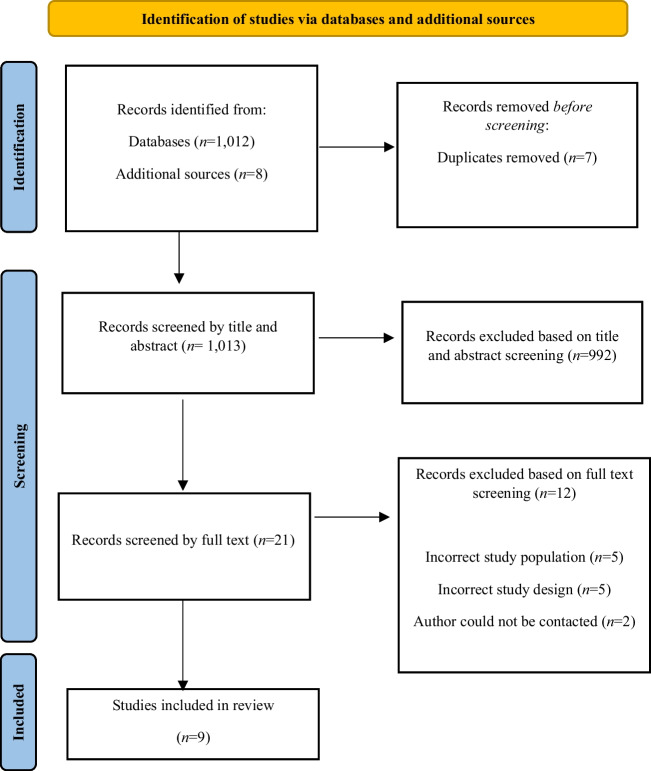
PRISMA flow diagram

Database searches returned 1012 articles, with an additional eight articles identified through secondary sources. Seven duplicates were removed leaving 1013 articles that were screened by title and abstract. Following title and abstract screening, 992 articles were removed leaving 21 articles to be screened by full text. Twelve did not meet the eligibility criteria following full-text screening. The primary reasons included incorrect study population (*n* = 5), incorrect study design (*n* = 5), and the authors could not be contacted (*n* = 2). A total of nine [30–38] articles met the pre-defined eligibility criteria and were included in the final analysis. A study flow diagram outlining the screening process and outcomes for this systematic review is reported in Fig. 1.

### Study characteristics

A total of nine studies were included in this review. Eight studies were conducted in Australia [30–33, 35–38] and one in New Zealand [34]. The number of rural lung cancer participants included across studies ranged from *n* = 1 to *n* = 70. Two studies included lung cancer participants alongside a range of cancer populations [30, 32], whereas six studies included lung cancer participants among other cancer types, healthcare professionals, carers, and family [31, 33–37]. Only one study focused exclusively on lung cancer patients [38]. All studies included rural, regional, or remote lung cancer populations, with four studies providing a comparison with non-rural populations [33, 36–38]. The majority of studies (*n* = 5) used solely qualitative designs [30, 31, 34, 35, 37] with four studies using mixed methods [32, 33, 36, 38]. Qualitative data were collected using semi-structured interviews [30–33, 35, 36, 38] with one study using focus groups [34] and another using interviews and focus groups [37]. Six studies defined rurality using a classification system [30, 32, 33, 35, 37, 38] whilst three studies did not report using a geographical classification system but did report conducting research in a rural, regional, or remote area [31, 34, 36]. For further details of study characteristics, see Table 1.

**Table 1:** Study characteristics

Study (country)	Population	Methods/design	Key findings
Crawford-Wiliams et al. [30] (2022) Australia Aim: To understand how cancer survivors in rural Queensland seek and receive information as well as their preferences regarding content and delivery of health-related information	Cancer patients *n* = 24 (lung cancer *n* = 6) Mean age = 63.8 years M/F = 11/13 Queensland, Australia Inner regional *n* = 9 Outer regional *n* = 9 Remote/very remote *n* = 6	Qualitative descriptive design using semi-structured interviews Reflective thematic analysis Postcodes geocoded and classified by remoteness of area according ASGS classification	Participants reported that health-related information provision was inconsistent and occasionally contradictory. Cancer survivors’ needs for information and attitudes towards seeking it varied greatly and many survivors had difficulty processing and retaining information due to emotional distress
Drury and Inma [31] (2010) Australia Aim: To explore patients’ experiences from perspectives of patients and healthcare providers in a regional area of Western Australia	Cancer patients *n* = 11 (lung cancer *n* = 2) Cancer nurse *n* = 1 Cancer support worker *n* = 3 Cancer educator *n* = 1 Age range = 40 to > 60 years M/F = 6/5 Regional Western Australia	Qualitative design using semi-structured interviews Thematic analysis used in an action research framework	Patients who had the involvement of a cancer nurse coordinator and cancer support workers reported better experiences and more streamlined care than did those who had to navigate the journey alone. It was suggested by most participants that metropolitan health professionals had little empathy or understanding of the travelling involved for rural patients
Emery et al. [32] (2013) Australia Aim: To compare and explore symptom appraisal and help-seeking behavior in patients with breast, lung, prostate, or colorectal cancer from rural Western Australia	Cancer patients *n* = 66 (lung cancer *n* = 8) Mean age = 60.5 years M/F = 28/38 Rural Western Australia	Mixed method design Qualitative data collected using semi-structured interviews Thematic framework analysis Rurality defined using ARIA	Differences were observed in symptom appraisal and help-seeking behaviors between patients with breast, lung, prostate, or colorectal cancer. Lung cancer patients reported being shocked with their lung cancer diagnosis and cited factors related to distance and stoicism that influenced longer symptom appraisal and help-seeking intervals
Hall et al. [33] (2008) Australia Aim: To investigate if the pattern of diagnostic testing for suspected lung cancer, stage at diagnosis, patterns of specialist referral, and treatment options offered to people in rural Western Australia are similar to those in the metropolitan area. To explore the barriers to quality care in rural areas as perceived by GPs and patients	Lung cancer patients *n* = 43 General practitioners *n* = 27 Mean age = 63.53 M/F = 26/17 Western Australia Rural *n* = 22 Metropolitan *n* = 21	Mixed method design Qualitative data collected using semi-structured interviews Interviews collected in *n* = 14 lung cancer patients only Grounded theory inductive analysis approach Rurality defined using ARIA	Rural patients perceived their symptoms as minor rather than cause to see their GPs. Only a quarter of rural patients expressed trust in their healthcare professionals. The quality of psychological care was an issue for rural patients. Rural patients were also less satisfied with aspects of their care, especially with the quality of communication, and some rural patients also expressed concerns about financial aspects of their care
Kidd et al. [34] (2021) New Zealand Aim: To explore the barriers to early presentation and diagnosis of lung cancer, as identified by Māori patients, whanau (families), and primary healthcare providers in Midland region of New Zealand	Community participants (lung cancer patients, family members and other community members) Healthcare providers (staff members including GPs and nurses)	Qualitative design using focus groups Nine focus groups conducted with both community participants and healthcare providers Thematic analysis Research was conducted in five rural localities in the Midland region of New Zealand	Māori lung cancer patients, family, and community members experienced long waiting times, poor communication across health services, and concerns as to how information was shared with them. The need for health services and professionals to better understand the cultural needs of Māori patients and family was highlighted as well as strategies that meet these needs. Family members had to take the initiative at various points in the patient’s cancer care journey, acting as enablers for patients
Otty et al. [35] (2023) Australia Aim: To explore the experiences and perceptions of people with lung cancer and their carers	Lung cancer patients *n* = 19 Lung cancer carers *n* = 7 Median age = 64 M/F = 10/9 Northern Australia Regional *n* = 12 Rural *n* = 4 Remote *n* = 3	Cross sectional descriptive qualitative study Qualitative data collected using semi-structured interviews Iterative inductive thematic analysis Rurality defined using MMA	Factors that impacted care experience were good communication, timeliness, patient advocacy, and support. Improper communication, long waiting times, uncertainty about the process, and inconsistent advice negatively impacted care experience
Page et al. [36] (2016) Australia Aim: To survey the level of lung cancer awareness in rural and remote Aboriginal and Torres Straight Islander communities and discover perceived barriers to timely diagnosis and treatment of lung cancer	Lung cancer patients *n* = 2 Community members *n* = 51 Health workers *n* = 14 Queensland, Australia Rural patient *n* = 1 Urban patient *n* = 1	Mixed method design Qualitative data collected using interviews Research conducted in outer regional and remote communities in addition to one urban setting	An Indigenous person diagnosed with lung cancer faces unique cultural, physical, and psychological challenges. The only rural patients reported delays in treatment and experienced financial hardship with poor access to culturally targeted information but were satisfied with the support and information provided. Lack of public transport was identified as a barrier to accessing health services in some communities
Rankin et al. [37] (2017) Australia Aim: To describe the lung cancer diagnostic pathway, focusing on the perspective of patients and general practitioners about diagnostic and pre-treatment intervals	Lung cancer patients *n* = 19 General practitioners *n* = 11 Mean age = 65 years M/F = 9/10 Sydney and New South Wales Metropolitan (63%) Inner regional (21%) Outer regional (16%)	Qualitative study design using in-depth interviews and a focus group Thematic content analysis using Mile’s and Huberman’s framework Rurality defined using ARIA	The lack of defined diagnostic pathways to respiratory specialist assessment and hospital clinics was frustrating for patients and GPs. Barriers (including travel) to accessing health services caused significant delays after receiving results, creating a sense of urgency. System factors were most salient for regional patients, as the geographical location of specialist providers and diagnostic services significantly impacted making appointments. Four of seven regional patients first presented to the emergency department without previously visiting their GP about symptoms or concerns
Verma et al. [38] (2018) Australia Aim: To identify any differences in time delays in lung cancer referral pathways between rural and urban patients and explore patients’ perceived barriers to timely lung cancer diagnosis	Lung cancer patients *n* = 252 Mean age = 65.2 M/F = 176/76 North Queensland Urban/Outer regional *n* = 182 Remote *n* = 70	Mixed method design Qualitative data collected using semi-structured interviews Thematic analysis Rurality defined using ASGC	Misinterpretation of symptoms was common among rural participants with some citing the reason of “not being a doctor person” for the increased delay in presenting to a GP. Several participants refused investigations causing further delays in diagnosis and management. Some rural participants perceived family commitments and work as more important than their own health. Travel and finance were regarded as barriers in their treatment and management

### Quality assessment

There was a low risk of bias across the majority of included studies [30, 32, 34, 35, 37]. Three of the studies gave vague details around the ethical approvals that were in place with no dates or ethics committee reference numbers [33, 36, 38]. The same three studies provided limited details surrounding data analysis [33, 36, 38]. Three studies [30, 34, 37] provided limited details surrounding the relationship between the researcher and participants whilst four studies failed to report on this at all [31, 33, 36, 38]. The results of the quality assessment are reported in Table 2.

**Table 2:**
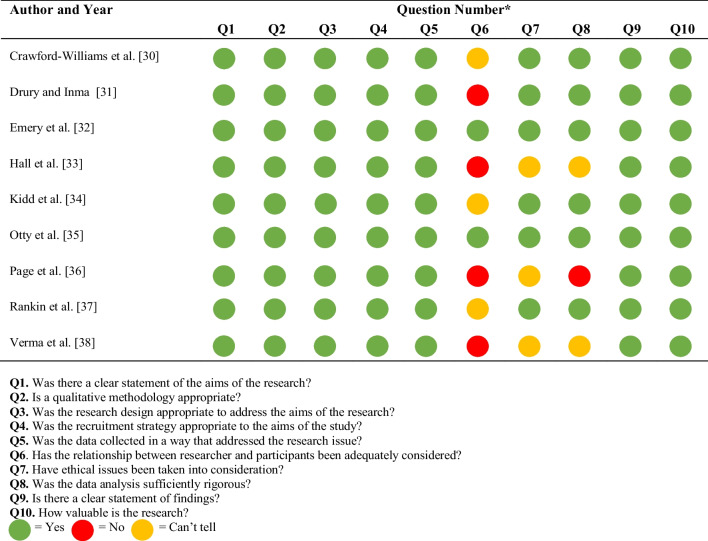
Quality assessment of included studies

### Thematic synthesis

A total of 50 initial codes were generated from all studies. These codes were grouped together based on similarities to form 18 descriptive themes. This led to the development of five analytical themes related to the experiences of people living with lung cancer who reside in rural areas. These included (1) diagnosis and treatment pathways, (2) travel and financial burden, (3) communication and information, (4) experiences of interacting with healthcare professionals, and (5) symptoms and health-seeking behaviors. Each analytical theme along with the descriptive themes and supporting verbatim quotations is presented in Table 3. A narrative account of the analytical themes is presented below.

**Table 3:** Analytical themes, descriptive themes, initial codes, and examples quotes from the qualitative analysis

Analytical theme and definition	Descriptive themes	Initial codes	Example quotes
Diagnosis and treatment pathways This theme relates to participants experiences of diagnosis and their treatment. For some, their diagnosis was unexpected. Participants reported frustrations with delays and long waiting times	**Unexpected diagnosis**	Shock of being diagnosed Sudden and unexpected diagnosis Surprise/potential misdiagnosis	“Yeah well I was shocked because um, you expect people who smoked and went out drinking and things like that, you know. Um, and you think if you look after yourself and be healthy and not being obese, ah, rather surprised me. I mean, if I was obese or something like that I would expect it I suppose” [32] “I mean my tumor was 6 cm when it was eventually diagnosed at the (metropolitan hospital) a week later. It could not grow just in a minute, it had to have been there for at least a long time…” [35]
	**Treatment delays**	Perceived unnecessary delays to treatment Long delays before initiating treatment Frustration in treatment delay	“With bronchoscopy – mucked around a bit, should have got on with treatment” [33] “And it was just near the end that I felt like, oh my god, please can we get something going, because I was really well aware of the fact there was really something there then, and I just wanted something to start” [35]
	**Poor follow-up support**	Abandonment after treatment Insufficient follow-up and support Felt let down after treatment	“Felt abandoned at end of treatment – back to GP” [33] “Then I got handed over to a number of other doctors. And so come November when it all finished, there was no indication of appointment follow-ups or scan or anything. And that’s where they fell down there a little bit” [35]
	**Long waiting times**	Unnecessary discharge wait Used to experiencing long waiting times Frustration in waiting for information	“Easy to get into each place and have treatment or tests, but getting clearance to leave often long overdue and waiting needlessly” [33] “You get used to the waiting, and although XXX Hospital very good it was a long day with the transport” [33]
	**Undiagnosed and misdiagnosis**	Misdiagnosis of lung cancer Undiagnosed lung cancer	“It was just the coughing up blood. Because I’ve got COPD anyway, I do have a little bit of breathlessness all the time, the doctor told me it was pneumonia.” [35] “I mean my tumor was 6 cm when it was eventually diagnosed at the (metropolitan hospital) a week later. It could not grow just in a minute, it had to have been there for at least a long time…” [35]
	**Patient advocates**	Family members acting as patient advocate Healthcare staff acting as patient advocate	“My brother, he actually took me on. Because I was too ill to email and fight for my rights so he took my email and started to say look when am I gonna get treatment? And he just happened to be rung to say we’re having this [PET] scan… And I was in there and he rung the oncologist and said my sister is in there now having a PET scan, please if you have a spare bed can she go in. And I went straight from there up, through my 1st round of chemo. But you have to fight as well for your treatment. And when you’re too ill, get someone who can talk to the pathologist or radiologist, to say when is it going to happen.” [34]
Travel and financial burden This theme relates to the travel and financial burden as a consequence of their lung cancer diagnosis. Hospitals in metropolitan urban centers were considered inaccessible and time consuming to get to. Having lung cancer also placed significant financial strain on the participants	**Travel burden**	Poor understanding of travel distance Long travel distances Adverse to travelling long distances for treatment Travel a barrier to accessing treatment	“I had to drive up to Perth to see the doctor and then 2 days later go up again for the start of treatment. They didn’t seem to understand the time and money” [31] “If I had to go to Brisbane for lung treatment, I would not go. Neither would others, especially Indigenous. Getting down to Brisbane is very hard” [36] After the biopsy was done I was back in (regional city) again to see (the respiratory physician). He got in touch with Professor (of radiation oncology) because he wanted us to go to Sydney (for treatment) and I said “well can’t we go to Orange?” It was so much easier for us.” [37]
	**Financial burden**	Poor understating of financial impact Financial worry Petrol and accommodation costs	“Now there is a problem with me getting to Townsville. My friend drove me last time and I still have not got any petrol money or accommodation money.”[38] “Great strain on finances” [33]
Communication and information This theme related to communication between health professionals and patients in relation to their lung cancer diagnosis and treatment. There were reports of poor communication, too much information, and a lack of information and examples of good communication from health professionals	**Poor communication**	Improved patient communication needed Difficulty in obtaining information Greater time needed for questions and understanding Impersonal communication approach	“Have to drag every bit of information out of the doctors – irritating them” [33] “Didn’t know initially which form of cancer it was and waiting to hear was very worrying” [33] “But in very broken English over a very bad phone line, I heard that – (1) I had lung cancer, (2) it had spread to my bones, hips, spine, lymph system; with treatment would be 12 months and I would be dead. Basically, the phone call was over in 15 min. Suddenly it went from I’m getting no information; I’m getting information that is unexpected, uncontextualized and not given in a format where I have the opportunity to understand or ask questions.” [35]
	**Poor information and awareness**	Poor self-management information Inadequate diagnosis and treatment information Unaware of supportive information	“When we were going through my chemo and radiotherapy … I lost a lot of weight, and we were looking for healthy ways to put on weight, and the recommendation at the hospital from the doctor was just go to McDonalds and have a chocolate thick shake. There’s no focus on quality nutrition.” [30] “did get information on the pill, which I still have. Um and I kind of understand what it’s doing.” [30]
	**Too much information**	Irrelevant and unnecessary information Overwhelmed with information	“I suppose because I do not know anything medical, do not want to see pictures, I just want to get on with it. I just want to get told what I have to do to get this over and done with’.” [35] “I think we received quite enough information. Like I think the only thing I wanted to know is, how much time have I got?” [35]
	**Good communications**	Good explanations and communication with patient Allowed time for patient to ask questions Adapting communicative skills	“The radiation oncologist lady was excellent. She talked you through things, she explained things, she gave you, gave you space to have an opinion or to ask questions” [30] “Happy with explanations given at radiation clinic, can understand it all: Scale of 1–10 = 10!” [33] “He was upfront, he said it me that it was that they had found a mass. He said a picture paints a thousand words, so he showed me my x-rays, and showed me how one third of my left lung had collapsed and showed me the mass that was on my lung.” [35]
Experiences of interacting with healthcare professionals This relates to people with lung cancer being dissatisfied with the healthcare professionals delivering their care. There were reports of a lack of sympathy and compassion. Finally, there were several participants who reported positive experiences of their interactions with healthcare professionals	**Dissatisfied with healthcare settings and professionals**	Poor attitude towards patient care Transparency in communicative behavior Impersonal nature of healthcare professionals Poor facilities and diagnostic/treatment centers	“but the medical oncologist at the [hospital] was disgusting.” [30] “Grave with care at AAA, nursing atrocious, attitude shocking and husband ignored; [need] better standard of nursing care” [33] “Worried when came back from Perth and in hospital again and hair falling out as no one had sent any paperwork to XXX for the next chemo treatment – specialist said not expected to live long enough to need it anyway!” [33]
	**Indifferent attitude**	Lack of empathy and compassion Arrogant and impersonable Insensitive nature	“When told diagnosis – lady could have been a bit more sympathetic” [33] “First time I had to deal with specialists, so have nothing to compare with but couldn’t stand the arrogance of those I saw” [33]
	**Satisfied with healthcare professionals**	Knowledgeable healthcare professionals Healthcare professionals attentive to care needs Provided adequate information Time taken to explain information Confidence in healthcare professional	“Excellent; feel doing everything possible and no stone unturned, confident because of it” [33] “Lots of time and trouble taken to explain things and I appreciated the booklet from the hospital” [33]
Symptoms and health-seeking behaviors The final theme relates to some rural people with lung cancer having traits of stoicism and being reluctant to ask for help especially due to their distance from acute treatment centers	**Reluctance to seek help**	Stoicism and sociocultural influences in seeking medical help Distance a barrier to seeking medical help	“And the country men are worse than the women, by a long shot. They’re, you know, bush blokes. You know, “I’m not going to the doctor. I’ll be right, mate**” **[32] And I’m thinking that, no this... this could get better without a trip into town. Because we... because we’re 40 k’s out, you think twice about coming in for every little cough and sniffle” [32]
	**Comorbidities**	Symptoms masked by co-existing conditions	“It was just the coughing up blood. Because I’ve got COPD anyway, I do have a little bit of breathlessness all the time” [35]
	**Caring responsibilities**	Prioritizing caring responsibilities over own health	“I am working hard looking after my handicapped son, that I did not pay attention to my cough, and that it had been worsening.” [38]

#### Diagnosis and treatment pathways

Participants expressed frustration in the delay in being diagnosed with lung cancer and the initiation of subsequent treatment with individuals suggesting having to wait months before receiving a formal diagnosis or beginning treatment [33, 35]. Some individuals had received an unexpected diagnosis [32, 35], with others suggesting that they were initially misdiagnosed and surprised at how their healthcare professional missed signs of lung cancer before being diagnosed [35]. Participants were dissatisfied with the long waiting times for results and treatment which they found frustrating and needless [33]. Participants alluded to a lack of choice as to where they received treatment suggesting that GP preference and those who received private medical cover were factors that minimized patient choice [33]. Participants emphasized the importance in having family members and even healthcare professionals that acted as patient advocates suggesting that they were integral in receiving timely information and coordinating treatment needs [34, 35]. Post-treatment, one participant expressed feeling abandoned and suggested having to revisit their GP for further information and support [33] whilst another participant experienced receiving no information regarding follow-up appointments or scans suggesting the healthcare team underperformed [35].

#### Travel and financial burden

Travelling to and from urban areas was viewed as a major barrier in seeking or receiving medical treatment [31–33, 36–38]. Some patients were reluctant to travel to urban areas at all due to the complexities of navigating long distances [31, 33, 36, 37] whereas others were mindful of travelling long distances to seek medical advice or treatment over minor symptoms [32]. Other patients suggested they would rather stop receiving treatment if travelling became too difficult [38]. For example, one participant suggested that if they had to receive treatment at a distant location that they would not go, and neither would others they knew, as travelling to these locations was perceived as challenging [36]. Another participant suggested that they were unsure about their upcoming trip and suggested that if it all became too hard that they might just let nature take its course [38]. Participants expressed feeling frustrated regarding the lack of understanding from healthcare professionals over the time, effort, and money required to travel to receive treatment [31]. Whilst finance was a worry for many individuals, the use of private medical cover reduced the stress associated with travel for some [33]. Several patients reported experiencing financial worry and stress in receiving treatment largely related to travel and accommodation [31, 33, 38].

#### Communication and information

Individuals reported poor communication from healthcare professionals related to their lung cancer diagnosis and treatment [33, 35]. Patients desired better communication from their healthcare professionals including enhanced explanations surrounding their diagnosis and treatment and more time to ask questions. One individual felt that they were irritating healthcare professionals by asking questions and felt that it was challenging to obtain information from healthcare professionals [33]. Others experienced receiving information about their diagnosis in an unexpected and contextualized manner with little opportunity to process the information or ask questions about the diagnosis [35]. One individual suggested that there was a poor focus on the quality of self-management information provided by healthcare professionals with respect to the nutrition needed to gain weight following treatment [30], whilst others explained that they were initially unaware of the type of cancer they had been diagnosed with [33], or any financial support available to them [33]. In some cases, patients were less interested in receiving information concerning their diagnosis but were more concerned with receiving information about potential treatment and disease prognosis [35]. Whilst poor communication and lack of information from healthcare professionals was problematic for some, others did report positive experiences regarding the communication and information provided by healthcare professionals [30, 33, 35]. For several individuals, information was explained clearly by their healthcare professional and opportunities were provided to express their opinion and ask any questions [30, 33]. One participant explained how their healthcare professional adapted their communication style to effectively communicate their diagnosis through use of pictures and x-rays rather than solely through words [35]. Others expressed their appreciation regarding their advice received on how to cope with being diagnosed with lung cancer as well as the resources provided from hospitals [33].

#### Experiences of interacting with healthcare professionals

Individuals’ experiences with healthcare professionals contrasted with some expressing dissatisfaction whilst others expressed positive experiences. Those who reported negative experiences were frustrated with the attitudes of healthcare professionals whilst receiving care, citing them as shocking, disgusting, and not forthcoming [30, 33, 35]. Others expressed disappointment with the lack of effort made to make them feel comfortable whilst in hospital. For example, one patient experienced nothing being offered in terms of food and drink [33]. A prominent concern expressed by patients was the lack of compassion from healthcare professionals during their diagnosis and treatment. Some patients were unhappy with the lack of sympathy regarding their diagnosis whilst others were frustrated with what was perceived by patients as a lack of compassion and arrogance of healthcare professionals [33]. On the other hand, patients did express positive experiences when interacting with healthcare professionals. Patients reported healthcare professionals to have been outstanding, knowledgeable, and practical with aspects of their treatment and support and felt that they were genuinely concerned for their well-being [33]. Some patients suggested that the support provided by healthcare professionals gave them confidence going forward [33].

#### Symptoms and health-seeking behaviors

Some did not recognize their symptoms of lung cancer due to perceiving them to be related to existing comorbidities [35]. Another participant reported having to engage in significant care responsibilities for family members suggesting that because of this they did not notice their potential symptoms of lung cancer worsening [38]. Some individuals living with lung cancer in rural areas showed traits of stoicism and appeared reluctant to seek help [32]. Some individuals simply did not want to visit a doctor with one participant suggesting that males living in rural areas known as “bush blokes” were perceived as particularly reluctant to seek help due to their stoic attitude whereas others were put off by the distance required to travel [32].

## Discussion

Globally, lung cancer is the second most common cancer [3] and this systematic review is novel in that it was the first to synthesize the qualitative academic evidence exploring the experiences of rural people living with lung cancer in OECD countries. Despite many OECD countries having large rural areas and populations, addressing cancer inequalities associated with residing in a rural area continues to be largely absent from health policy [21]. The wider existing literature explicitly reinforces that rural people living with cancer can experience unique care inequalities compared to their urban counterparts [12, 13]. Rurality is therefore a salient factor that merits urgent consideration by the lung cancer community. This review provides important insight on the individual experiences of rural people living with lung cancer, where much of the previous scientific activity in lung cancer has focused on the epidemiological and quality of clinical services.

Nine studies were included in this review from only two countries (Australia and New Zealand). The wider existing literature highlights that rural oncology research has been dominated by scholarly activity from North America and Australia [39–44] with an emerging body of survivorship research now coming from the UK [45–48]. Despite this, there were no European, North American, or UK-based studies included in this review indicating the need for further qualitative research within these geographic settings. That said, this review provides an important starting point in which the findings can be verified or challenged with additional high-quality research evidence in other OECD countries. The limited rural lung cancer research substantiates the need to reconceptualize the rural cancer research agenda as advocated by previous research [13, 49] through focusing on localized, community-based investigations that utilize qualitative and quantitative methods, as well as, co-production, to better capture the experiences and needs of rural people with lung cancer. This is markedly important in the context of the UK where there are currently three million people living with cancer [50] yet only limited research exists concerning the intersectionality between cancer and rurality. There are a significant number of people living with lung cancer residing in rural areas who likely face unique challenges related to travel, finances, and access [44]. It is important that rural coastal areas are not neglected either as they are typically characterized by high levels of deprivation, alcohol abuse, smoking, and poor physical and mental health [51] that may impact on lung cancer risk. This is particularly evident within the UK in which there have been recent calls from the UK government for a national strategy to improve the health and well-being of coastal communities [52].

Difficulty in accessing cancer services was reported by rural people with lung cancer, largely related to significant travel distances and financial constraints. This is evidenced across the wider cancer survivorship literature [53, 54], where the lack of available specialist treatment centers and support services [55, 56] combined with poor recruitment and retention of highly skilled healthcare professionals [57, 58] are underlying factors that exacerbate poor accessibility experienced by rural communities. The inaccessibility of readily available treatment has significant implications for disease outcomes with greater travel distance being associated with more advanced disease at diagnosis, inadequate treatment, poorer disease prognosis, and worse quality of life [59]. These issues may also be compounded by sociocultural factors (e.g., attitudes, beliefs, societal norms) that may dissuade rural communities from seeking help [60]. This was evident in the current review where individuals suggested that they avoided seeking medical help due to factors such as travel distance, socialcultrial beliefs, and the prioritization of their work and family commitments. It is paramount that more equitable access to cancer services is provided for rural people with lung cancer that addresses travel distance and its financial impact as well as the sociocultural factors that may prevent individuals from seeking treatment. Mobile screening and detection services [61, 62] as well as telemedicine [63, 64] are two proactive and innovative approaches that should be considered a focal point of future strategies to mitigate travel and financial barriers, provide outreach and education, and improve rural cancer outcomes.

Rural people living with lung cancer in our review reported being surprised with their diagnosis and the progression of the disease at the time of diagnosis. This is widely reported across the existing literature as lung cancer can often be difficult to diagnose early [65]. Long treatment delays and waiting times were also two prominent findings in the current review. Delays in cancer treatment are a global issue, in which a recent meta-analysis suggests that even a 4-week delay in treatment (surgery, systemic treatment, or radiotherapy) is associated with a significant increase in lung cancer mortality [66]. Greater efforts are therefore needed to address system level treatment delays to improve lung cancer survival following diagnosis. However, it is important to note that longer treatment delays are observed in less symptomatic lung patients but typically associated with better disease prognosis [67]. Some participants in the current review also experienced poor follow-up support from healthcare professionals and services post-lung cancer treatment. Cancer patients are often faced with a range of physical and psychosocial challenges post-treatment in which the support provided by clinicians is rated poorly [68]. Improved awareness is needed by healthcare professionals surrounding the support needs of lung cancer patients post-treatment in addition to greater signposting to professional, community, and voluntary organizations who may provide tailored support for lung cancer patients.

The poor communication of information from healthcare professionals was another issue identified in this review that reflects the wider experiences of people living with cancer [69]. Many people living with lung cancer experience uncertainty about their diagnosis and prognosis and are unclear about management and treatment plans [70]. Consequently, poor communication and information can have a detrimental impact upon the management of symptoms, treatment decisions, psychosocial health, and overall quality of life [71, 72], indicating the need to introduce more practical efforts to improve the communication of information between the patient and healthcare system in addition to the communicative skills of individual healthcare professionals. Furthermore, the quality and amount of information provided to patients was highlighted as problematic in this review. Health literacy (i.e., the skills, knowledge, understanding and confidence to access, comprehend, and use information) should be an important consideration when communicating and providing information. Evidence suggests that cancer outcomes may be poorer for those who experience difficulty understanding information or who are overloaded with information [73]. Greater efforts must be made by healthcare professionals to understand how patients process information and how they use information to make decisions about their treatment and care.

We acknowledge several limitations as part of this research. Firstly, the included studies and findings are entirely drawn from an Australasian perspective. We recognize that the restricted geographic scope limits the international generalizability of our findings, and thus we strongly advocate for further qualitative investigations to examine and assess the applicability of our findings in the context of other geographical settings. However, findings from this study may hold great importance for people living with lung cancer in rural, regional, and remote areas of Australia. Approximately 7 million people (28% of the Australian population) reside in outer regional, rural, or remote areas spread across a large geographical area [74]. Our findings contribute towards a better understanding of the experiences and challenges of people living with lung cancer in rural Australia which could be used to better support researchers and healthcare providers in developing tailored services and interventions that lead to more personalized and patient-centered care in these settings. Secondly, this review is wholly focused on providing a patient-centered perspective of living with lung cancer in rural areas in which we acknowledge that the omission of carer and healthcare professionals’ perspectives as a limitation. Integrating the experiences of carers and healthcare professionals alongside people with lung cancer’s perspectives could enhance our understanding of lung cancer care in rural areas and augment the potential for the practical implementation of targeted interventions and support strategies. Thirdly, whilst we employed a rigorous and systematic approach to identify appropriate evidence, there were relevant qualitative studies that were excluded as they did not provide adequate detail to differentiate between geographical location or tumor site. We strongly encourage future studies to ensure that data is collected and presented with greater transparency to allow researchers to distinguish between study population groups. Furthermore, we recognize that certain themes (e.g., experiences of interacting with healthcare professionals) as well as sub-themes (e.g., long waiting times, indifferent attitude, and satisfied with healthcare professionals) rely heavily on the findings on a single paper from 2008 [28]. We acknowledge this as a limitation of the review and suggest that these findings are interpreted with caution. The inclusion of an individual with lived experience of caring for someone with lung cancer (AH-B) as a member of the research team greatly enhanced the review through providing unique perspectives that helped interpret and contextualize the study findings. However, we recognize the omission of people with lived experience of lung cancer when conducting this systematic review and we strongly recommend that future studies include both people with lung cancer and their carers where appropriate. Finally, the included studies in this review were deemed to be of moderate–high quality. However, future research efforts should prioritize more transparent reporting practices especially surrounding author reflexivity and the relationship between the authors and the participants.

This review has several potential clinical implications for health professionals supporting rural people with lung cancer. Support in accessing high-quality diagnostic and treatment services may be important with timely and clear communication of information regarding patient illness and the services which will treat and care for individuals. Lung cancer care should be provided by structured teams with integrated care across the various healthcare sectors [75, 76], with a focus on quality of life, survival, integrated palliative care services, and access to research, clear survivorship policies [77], and information [78, 79]. Healthcare systems should consider greater training and support for healthcare professionals [80, 81] to better engage with lung cancer patients. The use of cancer care coordinators could be a potential solution as part of future strategies to help improve care co-ordination, navigate complex healthcare systems, facilitate enhanced communication, and signpost to appropriate resources and support services [82]. Clearer cancer awareness campaigns should be considered to place greater emphasis on lung cancer screening, education, and treatment pathway awareness in rural areas [83]. Furthermore, greater support could be provided, for example, by governments and healthcare organizations, to reduce the financial and travel burden placed on rural lung cancer patients as well as close family and friends [84]. In doing so, this could potentially facilitate improved early detection and screening uptake, better patient access to specialized cancer services, and ensure timely and continuous treatment for rural lung cancer patients. Whilst support services (e.g., financial, psychological, and transport) are already available in some countries (e.g., the UK), they vary regionally and are often underutilized highlighting the need for greater awareness for these services. Finally, although many individuals express preference for face-to-face appointments, the use of telemedicine should be considered to provide remote care and support to help negate the financial and travel barriers placed upon individuals living in rural areas. Telemedicine has the potential to revolutionize cancer care [85], especially in areas where healthcare resources are limited, and should be used as a complementary tool as part of cancer care [63].

## Conclusion

This systematic review is the first to synthesize the qualitative academic evidence surrounding the experiences of rural people living with lung cancer in OECD countries. Addressing cancer inequalities associated with residing in a rural area continues to be mostly absent from international policy. The findings of this review enable a deeper understanding of the issues faced by people with lung cancer in rural areas, through which future researchers could develop tailored support to better address the existing health disparities that they may face. Additionally, this study provides an important starting point in which the findings can be verified or challenged through further high-quality evidence across other geographical settings.

### Supplementary Information

Below is the link to the electronic supplementary material.

Supplementary file1 (DOCX 28.4 KB)

Supplementary file2 (DOCX 31.2 KB)

Supplementary file3 (DOCX 25.1 KB)

## Data Availability

All data supporting the findings of this research are available within the article or its supplementary material.
