# Supplementary material for: A systematic review on the qualitative experiences of people living with lung cancer in rural areas
Source: Support Care Cancer. 2024 Feb 6;32(3):144. doi: 10.1007/s00520-024-08342-4 (PMC10844412; doi:10.1007/s00520-024-08342-4)
Supplement: Supplementary file 1 — Supplementary file1 (DOCX 28.4 KB) [file 520_2024_8342_MOESM1_ESM.docx]

**A systematic review on the qualitative experiences of people living with lung cancer in rural areas**

Nabilah Ali^1^, David Nelson^2,3^, Daisy McInnerney^4^, Samantha L Quaife^4^, Despina Laparidou^5^, Peter Selby^6,1^, Ros Kane^7^, Sarah Civello^8^, Dawn Skinner^9^, Zara Pogson^8^, Michael D. Peake^10,11^, Ava Harding-Bell^12^, Samuel Cooke^2^

^1^ Lincoln Medical School, College of Health and Science, Universities of Nottingham and Lincoln, Lincoln LN6 7TS, UK.

^2^ Lincoln International Institute for Rural Health, College of Health and Science, University of Lincoln, Lincoln LN6 7TS, UK.

^3^ Macmillan Cancer Support, London SE1 7UQ, UK.

^4^ Centre for Cancer Screening, Prevention and Early Diagnosis, Wolfson Institute of Population Health, Queen Mary University of London, London, EC1M 6BQ, UK.

^5^ Community and Health Research Unit, School of Health and Social Care, University of Lincoln, Lincoln LN6 7TS, UK.

^6^ School of Medicine, University of Leeds, Leeds LS2 9JT, UK.

^7^ School of Health and Social Care, University of Lincoln, Lincoln LN6 7TS, UK.

^8^ Lincoln County Hospital, United Lincolnshire Hospitals NHS Trust, Lincoln LN2 5QY, UK.

^9^ Pilgrim Hospital, United Lincolnshire Hospitals NHS Trust, Boston, PE21 9QS, UK.

^10^ Cancer Research UK, London, E20 1JQ, United Kingdom

^11^ Glenfield Hospital, University of Leicester, LE1 7RH, UK

^12^ Swineshead Patient Participation Group, Swineshead Medical Group, Boston, Lincolnshire, PE20 3JE, UK

Corresponding author: Dr Samuel Cooke, Lincoln International Institute for Rural Health, College of Health and Science, University of Lincoln, Lincoln LN6 7TS, UK.. Email: [scooke@lincoln.ac.uk](mailto:scooke@lincoln.ac.uk) ORCID: [0000-0002-3027-7807](https://orcid.org/0000-0002-3027-7807)

**Journal:** Supportive Care in Cancer

Appendix 1 – ENTREQ checklist

| No | Item | Guide and description | Location |
| --- | --- | --- | --- |
| 1 | Aim | State the research question the synthesis addresses. | Page 3/4 – Introduction |
| 2 | Synthesis methodology | Identify the synthesis methodology or theoretical framework which underpins the synthesis and describe the rationale for choice of methodology (e.g. meta-ethnography, thematic synthesis, critical interpretive synthesis, grounded theory synthesis, realist synthesis, meta-aggregation, meta-study, framework synthesis). | Page 5 – Data analysis |
| 3 | Approach to searching | Indicate whether the search was pre-planned (comprehensive search strategies to seek all available studies) or iterative (to seek all available concepts until they theoretical saturation is achieved). | Page 4 – Study design – Protocol registration |
| 4 | Inclusion criteria | Specify the inclusion/exclusion criteria (e.g. in terms of population, language, year limits, type of publication, study type). | Page 4/5 – Eligibility criteria |
| 5 | Data sources | Describe the information sources used (e.g. electronic databases (MEDLINE, EMBASE, CINAHL, psycINFO, Econlit), grey literature databases (digital thesis, policy reports), relevant organisational websites, experts, information specialists, generic web searches (Google Scholar) hand searching, reference lists) and when the searches conducted; provide the rationale for using the data sources. | Page 4 – Search strategy |
| 6 | Electronic search strategy | Describe the literature search (e.g. provide electronic search strategies with population terms, clinical or health topic terms, experiential or social phenomena related terms, filters for qualitative research, and search limits). | Supplementary Information 3 |
| 7 | Study screening methods | Describe the process of study screening and sifting (e.g. title, abstract and full text review, number of independent reviewers who screened studies). | Page 4 – Search Strategy |
| 8 | Study characteristics | Present the characteristics of the included studies (e.g. year of publication, country, population, number of participants, data collection, methodology, analysis, research questions). | Pages 8 – Study Characteristics  Table 1 |
| 9 | Study selection results | Identify the number of studies screened and provide reasons for study exclusion (e,g, for comprehensive searching, provide numbers of studies screened and reasons for exclusion indicated in a figure/flowchart; for iterative searching describe reasons for study exclusion and inclusion based on modifications t the research question and/or contribution to theory development). | Page 6 – Results  Figure 1 |
| 10 | Rationale for appraisal | Describe the rationale and approach used to appraise the included studies or selected findings (e.g. assessment of conduct (validity and robustness), assessment of reporting (transparency), assessment of content and utility of the findings). | Pages 5– Quality assessment |
| 11 | Appraisal items | State the tools, frameworks and criteria used to appraise the studies or selected findings (e.g. Existing tools: CASP, QARI, COREQ, Mays and Pope [25]; reviewer developed tools; describe the domains assessed: research team, study design, data analysis and interpretations, reporting). | Pages 5 – Quality assessment |
| 12 | Appraisal process | Indicate whether the appraisal was conducted independently by more than one reviewer and if consensus was required. | Pages 5– Quality assessment |
| 13 | Appraisal results | Present results of the quality assessment and indicate which articles, if any, were weighted/excluded based on the assessment and give the rationale. | Page 11 – Quality assessment  Table 2 |
| 14 | Data extraction | Indicate which sections of the primary studies were analysed and how were the data extracted from the primary studies? (e.g. all text under the headings “results /conclusions” were extracted electronically and entered into a computer software). | Page 5 – Data extraction  Page 5 – Data analysis |
| 15 | Software | State the computer software used, if any. | Page 4 – Search strategy  Page 5 – Data analysis |
| 16 | Number of reviewers | Identify who was involved in coding and analysis. | Page 5 – Data analysis |
| 17 | Coding | Describe the process for coding of data (e.g. line by line coding to search for concepts). | Page 5– Data analysis |
| 18 | Study comparison | Describe how were comparisons made within and across studies (e.g. subsequent studies were coded into pre-existing concepts, and new concepts were created when deemed necessary). | Page 5 – Data analysis  Table 3 |
| 19 | Derivation of themes | Explain whether the process of deriving the themes or constructs was inductive or deductive. | Page 5 – Data analysis |
| 20 | Quotations | Provide quotations from the primary studies to illustrate themes/constructs and identify whether the quotations were participant quotations of the author’s interpretation. | Table 3 |
| 21 | Synthesis output | Present rich, compelling, and useful results that go beyond a summary of the primary studies (e.g. new interpretation, models of evidence, conceptual models, analytical framework, development of a new theory or construct). | Pages 11 - 13 – Thematic synthesis  Pages 18 - 20 – Discussion |
