# Supplementary material for: A systematic review on the qualitative experiences of people living with lung cancer in rural areas
Source: Support Care Cancer. 2024 Feb 6;32(3):144. doi: 10.1007/s00520-024-08342-4 (PMC10844412; doi:10.1007/s00520-024-08342-4)
Supplement: Supplementary file 3 — Supplementary file3 (DOCX 25.1 KB) [file 520_2024_8342_MOESM3_ESM.docx]

**A systematic review on the qualitative experiences of people living with lung cancer in rural areas**

Nabilah Ali^1^, David Nelson^2,3^, Daisy McInnerney^4^, Samantha L Quaife^4^, Despina Laparidou^5^, Peter Selby^6,1^, Ros Kane^7^, Sarah Civello^8^, Dawn Skinner^9^, Zara Pogson^8^, Michael D. Peake^10,11^, Ava Harding-Bell^12^, Samuel Cooke^2^

^1^ Lincoln Medical School, College of Health and Science, Universities of Nottingham and Lincoln, Lincoln LN6 7TS, UK.

^2^ Lincoln International Institute for Rural Health, College of Health and Science, University of Lincoln, Lincoln LN6 7TS, UK.

^3^ Macmillan Cancer Support, London SE1 7UQ, UK.

^4^ Centre for Cancer Screening, Prevention and Early Diagnosis, Wolfson Institute of Population Health, Queen Mary University of London, London, EC1M 6BQ, UK.

^5^ Community and Health Research Unit, School of Health and Social Care, University of Lincoln, Lincoln LN6 7TS, UK.

^6^ School of Medicine, University of Leeds, Leeds LS2 9JT, UK.

^7^ School of Health and Social Care, University of Lincoln, Lincoln LN6 7TS, UK.

^8^ Lincoln County Hospital, United Lincolnshire Hospitals NHS Trust, Lincoln LN2 5QY, UK.

^9^ Pilgrim Hospital, United Lincolnshire Hospitals NHS Trust, Boston, PE21 9QS, UK.

^10^ Cancer Research UK, London, E20 1JQ, United Kingdom

^11^ Glenfield Hospital, University of Leicester, LE1 7RH, UK

^12^ Swineshead Patient Participation Group, Swineshead Medical Group, Boston, Lincolnshire, PE20 3JE, UK

Corresponding author: Dr Samuel Cooke, Lincoln International Institute for Rural Health, College of Health and Science, University of Lincoln, Lincoln LN6 7TS, UK.. Email: [scooke@lincoln.ac.uk](mailto:scooke@lincoln.ac.uk) ORCID: [0000-0002-3027-7807](https://orcid.org/0000-0002-3027-7807)

**Journal:** Supportive Care in Cancer

Appendix 3 – Search strategy

| **Search Terms** |
| --- |
|  |
| **Lung Cancer Terms** |
| "Lung neoplasms" |
| “Lung cancer” |
| “Lung carcinoma” |
| “Small cell lung carcinoma” |
| “Non-small cell lung cancer” |
| “Non-small cell lung carcinoma” |
|  |
| *"Lung neoplasms" OR “Lung cancer” OR “Lung carcinoma” OR “Small cell lung carcinoma” OR “Non-small cell lung cancer” OR “Non-small cell lung carcinoma”* |
|  |
| **Rural terms** |
| Rural |
| “Rural population*” |
| “Rural area*” |
| “Rural communit*” |
| Nonurban |
| “Non-urban” |
| Remote |
| Regional |
| Isolated |
| “Small town*” |
| Village*  Settlement* |
| *Rural OR “Rural population” OR “Rural area” OR “Rural communit*” OR Nonurban OR Non-urban OR Remote OR Regional OR nonurban OR Isolated OR “Small town*” OR Village* OR Settlement** |
|  |
| **Experiences Terms** |
| Experience* |
| Perception* |
| Perspective* |
| Attitude* |
| Opinion* |
| View* |
| *Experience* OR Perception* OR Perspective* OR Attitude* OR Opinion* OR View**  **Lung cancer terms AND Rural terms AND Experiences terms** |
|  |
| *"Lung neoplasms" OR “Lung cancer” OR “Lung carcinoma” OR “Small cell lung carcinoma” OR “Non-small cell lung cancer” OR “Non-small cell lung carcinoma” AND Rural OR “Rural population” OR “Rural area” OR “Rural communit*” OR Nonurban OR Non-urban OR Remote OR Regional OR nonurban OR Isolated OR “Small town*” OR Village* OR Settlement* AND Experience* OR Perception* OR Perspective* OR Attitude* OR Opinion* OR View** |
